# Disease Germs

**Published:** 1872-03

**Authors:** I. N. Danforth

**Affiliations:** Chicago


ARTICLE II.—*Disease Germs.* By I. N. DANFORTH, M.D., Chicago.

FIRST PAPER.

Whatever the ultimate fate of the so-called "germ theory" of disease may be, certain it is that it has, within the past five years, chiefly through the labors of Lister, Tyndall and Beale, deeply stamped itself upon the thought, and largely influenced the practice, of the profession. Although the germ theory has of late assumed a more tangible and less speculative form, and has in consequence considerably modified former modes of practice, it is not by any means a theory of our day exclusively. Kircher, many years ago, advanced the opinion, "that epidemic diseases are due to germs which float in the atmosphere, enter the body, and produce disturbance by the development within the body of parasitic life;" and this idea found favor with the eminent naturalist Linnæus. While the germ theory was still struggling for a place in the minds of physicians and surgeons, and was by the great majority of them scouted as a fantastic dream, Sir Henry Holland expressed his belief that "some form of this theory was probably true."

For my own part, it requires no stretch of my imagination to believe that very minute particles of living matter (germs) may float in the atmosphere, may find entrance into the system, may therein proceed to multiply, and thus become fruitful sources of disease. Probably, however, the majority of physicians are not yet ready to assent to the germ theory, in all its length and breadth. The idea of such multitudes of parasitic tenants seems, at first sight, revolting and unendurable; yet it is no more revolting than the vague and unsatisfactory fermentation theory, which occasions changes in the blood, says Liebig, "just such as occur in beer making."* It is, however, a matter of considerable moment what one's belief is, since his practice must inevitably be modified thereby, and, chiefly for that reason, I wish to direct the attention of the readers of the JOURNAL to some considerations which seem to me to point towards the truthfulness of the germ theory.

I. The ingenious and beautiful experiments of Prof. Tyndall seem to demonstrate that the millions of dust particles, which we

* Simon's General Pathology, page 203.

all know so constantly float in the air we breathe, are not merely particles of finely powdered inorganic matter, but that they consist largely of organic atoms. Prof. Tyndall found that when the air was made to pass through a cold platinum tube, containing a roll of platinum gauze, it came forth "full of particles;" but when the tube and the gauze within it were heated to redness, the air issued from it "optically empty," that is, the floating matter "totally disappeared."* Hence these particles must be of organic origin, since inorganic matter is not so completely combustible. Having thus established the organic nature of these atoms, the question next arises, *what* are they? Are they living germs, capable of self-propagation, or merely floating particles of dead matter? The answer is, that they are partly living and partly dead; when living, they are capable of practically limitless self-multiplication whenever they chance to alight upon favorable soil; when dead, they are probably inert, or, at the worst, merely irritants which bathing, or an increased flow of mucus, with its accompanying cough or sneeze, will at once dislodge. Prof. Tyndall finds this dust to consist of "particles of ground straw, torn rags, smoke, the pollen of flowers, the spores of fungi and the germs of other things."† The experiments of Tyndall were made in London, where we should, of course, expect to find the atmosphere thickly laden with impurities.

On the other hand, Dr. Maddox has made many microscopic examinations of particles collected by means of his ingenious apparatus (figured in Beale's "Disease Germs,") from the atmosphere of Woolstone, near Southampton, which, he concludes, "cannot be considered as *loaded* with microscopic germs; the largest number visible and counted as such on one cover being twenty-one, (not including bacteroid bodies.")‡ But if twenty-one germs, exclusive of the possibly equally potent bacteroids, lodged upon a single glass cover, measuring, probably, three-quarters of an inch in diameter; and if each one of these were capable of the marvellous growth which usually distinguishes the germs of microscopic beings, then we must conclude the purest air is always thickly

* Nature, vol. I, page 340.

† Braithwaite's Retrospect, part lxiv, page 29.

‡ Beale's "Disease Germs; their Real Nature," page 79.

populated with these minute particles, whatever the fact may be concerning their power for evil.

II. In the next place it is an additional argument in favor of the germ theory, that recent modes of practice which have been adopted with the view of destroying or excluding germs, have proved more satisfactory in their results, than the most approved methods based upon other ideas. In regard to this, Prof. Lister speaks without doubt or hesitancy. In 1867, in an address delivered before the "British Medical Association," on the "Antiseptic Principle in the Practice of Surgery," he strongly advocates the constant use of carbolic acid in the treatment of wounds, ulcers, and all granulating surfaces, as well as in the dressing of compound fractures. It appears, he says, "to exercise a peculiarly destructive influence upon low forms of life, and hence is the most powerful antiseptic with which we are at present acquainted." In the treatment of compound fractures, Prof. Lister attaches great importance to the exclusion or destruction of "septic germs," which constantly produce decomposition of the purulent discharge. In the management of abscesses and sinuses, continues he, "all that is necessary is to guard against the introduction of *living atmospheric germs* from without, at the same time that free opportunity is afforded for the escape of discharge from within."* As regards the practical results of this mode of treatment, Mr. Lister says, "during the last nine months not a single case of pyæmia, hospital gangrene or erysipelas has occurred;" although previously he "felt ashamed when recording the results of his practice, to have so often to allude to these diseases."† One very interesting exceptional case is recorded by Mr. Lister; an abscess, located near the colon was opened, and in spite of the protection of the incision by carbolic acid dressings, the pus became extremely offensive, and the microscope detected the presence of myriads of vibrios. Death ensued, and the post mortem examination revealed the fact that the abscess communicated with the colon, from whence, therefore, the germs must have been derived. In a still later essay upon this subject, Lister expresses his unshaken belief that "putrefaction under atmospheric influence, as it occurs in surgical practice, is due to

* Ranking's Abstract, vol. xlvi, fol. 148.

† Op. cit., page 149.

particles of dust ever present in the atmosphere that surrounds our patients, and endowed with wonderful chemical energy and power of self-propagation, yet happily readily deprived of energy by various agents, which may be employed for the purpose without inflicting serious injury upon the human tissues."*

The wonderful experiments, of Prof. von Recklinghausen, of Wurzburg, upon blood, have a direct bearing upon this question of the agency of floating particles in producing putrefaction. Mr. Geo. Henry Lewis, writing to Prof. Tyndall, says : " Last spring, when I was at his (Recklinghausen's) laboratory in Wurzburg, I examined, with him, blood that had been three weeks, a month, and five weeks, out of the body, preserved in little porcelain cups under glass shades. This blood was living and growing. Not only were the ameba-like movements of the white corpuscles present, but there were abundant evidences of the growth and development of the corpuscles. I also saw a frog's heart still pulsating, which had been removed from the body (I forget how many days, but certainly more than a week.) There were other examples of the same persistence of vitality or absence of putrefaction. Von Recklinghausen did not attribute this to the absence of germs—germs were not mentioned by him; but when I asked him how he represented the thing to himself, he said the whole mystery of his operation consisted in keeping the blood *free from dirt.*"†

It is noticeable that Von Recklinghausen substitutes the term " dirt " for " germs ; " the practical difference, however, is simply *nothing ;* since, if his specimens were exposed to the air, they would shortly be peopled with forms of low life derived from germs. The interesting results of Prof. von Recklinghausen's experiments —whatever his theory concerning dirt or germs may be—aid us considerably in understanding the satisfactory consequences of the mode of treatment which Prof. Lister so ardently praises.

This subject will be continued in a future paper.

* Braithwaite, for January, 1872.

† Braithwaite, for January, 1872, p. 29.

